# Osteoporosis Prediction Using Machine-Learned Optical Bone Densitometry Data

**DOI:** 10.1007/s10439-023-03387-8

**Published:** 2023-10-26

**Authors:** Kaname Miura, Shigeo M. Tanaka, Chanisa Chotipanich, Thanapon Chobpenthai, Attapon Jantarato, Anak Khantachawana

**Affiliations:** 1 Biological Engineering Program, King Mongkut’s University of Technology Thonburi, Bangkok, 10140 Thailand; 2 Graduate School of Natural Science and Technology, Kanazawa University, Kanazawa, 920-1192 Japan; 3 Institute of Science and Engineering, Kanazawa University, Kanazawa, 920-1192 Japan; 4 National Cyclotron and PET Center, Chulabhorn Hospital, Bangkok, 10140 Thailand; 5 Faculty of Medicine and Public Health, HRH Princess Chulabhorn College of Medical Science, Chulabhorn Royal Academy, Bangkok, 10140 Thailand; 6 Department of Mechanical Engineering, King Mongkut’s University of Technology Thonburi, Bangkok, 10140 Thailand

**Keywords:** Osteoporosis screening, Machine learning, Optics, Demographic data

## Abstract

Optical bone densitometry (OBD) has been developed for the early detection of osteoporosis. In recent years, machine learning (ML) techniques have been actively implemented for the areas of medical diagnosis and screening with the goal of improving diagnostic accuracy. The purpose of this study was to verify the feasibility of using the combination of OBD and ML techniques as a screening tool for osteoporosis. Dual energy X-ray absorptiometry (DXA) and OBD measurements were performed on 203 Thai subjects. From the OBD measurements and readily available demographic data, machine learning techniques were used to predict the T-score measured by the DXA. The T-score predicted using the Ridge regressor had a correlation of *r* = 0.512 with respect to the reference value. The predicted T-score also showed an AUC of 0.853 for discriminating individuals with osteoporosis. The results obtained suggest that the developed model is reliable enough to be used for screening for osteoporosis.

## Introduction

One of the priorities for the treatment and prevention of osteoporosis is the early identification of individuals with low bone mineral density (BMD) [40]. Osteoporosis is a bone disease that decreases bone strength and increases the risk of fracture [32]. Bone strength is determined by two factors: BMD, which is a measure of bone mass, and bone quality, which consists of bone structure and microfractures, with BMD explaining approximately 70% of bone strength [32]. Bone mass increases during growth and reaches peak bone mass in the 20 s. Thereafter, it remains relatively stable and then declines with age [14]. Women, in particular, are prone to bone loss with menopause. Fractures due to osteoporosis reduce life function and quality of life [13, 33], and in the long term, osteoporosis, with or without fractures, significantly increases the risk of mortality [8, 31]. Therefore, its countermeasure is an important issue not only in medicine but also in society. In many cases, however, bone loss is not accompanied by subjective symptoms [12]. In addition, osteoporosis is difficult to treat with pharmacotherapy unless early intervention is provided [32]. Thus, early detection of individuals with low BMD can be an effective tool for early intervention of osteoporosis.

Currently, the gold standard for bone densitometry is dual-energy x-ray absorptiometry (DXA), which is used to diagnose osteoporosis [19, 7, 32]. However, DXA-based osteoporosis screening has not been widely used in many countries and situations because of its high cost, lack of portability, and potentially harmfulness. For people in developing countries, including Thailand, especially those living in suburban and rural areas, it is inconvenient to access DXA in community health service centers [36, 37, 42]. In addition to DXA, other X-ray-based bone densitometry methods exist, such as quantitative computed tomography (QCT), radiographic absorptiometry (RA), and digital X-ray radiogrammetry (DXR), all of which are less convenient for early screening for the same reasons as DXA. QUS, on the other hand, is the only method to measure bone without ionizing radiation and is used as an osteoporosis screening instruments. However, QUS is not sufficiently accurate to identify individuals with low BMD [23, 30]. QUS also requires the application of gel to match the acoustic impedance, increasing the total time required for measurement. Considering the limitations of these methods, there is a need for a compact, safe, and reliable pre-screening method to identify individuals with low BMD.

To achieve compact, safe, and reliable screening of osteoporosis, bone densitometry using near-infrared light has been studied as a new noninvasive method of BMD assessment [5, 24, 25]. Near-infrared light has excellent biological transparency [1], and there is a strong relationship between BMD and light scattering phenomena [43, 45]. Near-infrared light is significantly scattered by bone due to variations in the refractive index associated with hydroxyapatite and morphological changes [43]. In addition, a linear correlation exists between light scattering coefficient and BMD [45]. We previously developed an optical bone densitometry (OBD) method using a simple optical consisting of a laser diode module, a photodiode, two plano-convex lenses, and two annular slits [24, 25]. The OBD measures the change in light scattering caused by differences in BMD as the slope of the light intensity distribution formed when a near-infrared laser beam is irradiated on the skin surface [24]. Phantom experiments and numerical simulations for OBD showed that the slope of the light intensity distribution, which is formed on the skin's surface when irradiated with near-infrared light, correlated with BMD. The OBD could be developed into a portable device that facilitates screening of the general population for osteoporosis, and its small size makes it convenient when portability is required.

Even if sufficient measurement accuracy is confirmed in the laboratory, noninvasive medical measurement devices may not have sufficient screening performance when measuring humans, due to complex structure of tissues and systematic bias. For example, in the case of QUS, Evans and Tavakoli demonstrated a highly significant correlation (*r* = 0.85) between bovine femur velocity and physical density [9]. However, many in vivo validations show only moderate correlations between sound velocity and BMD [30, 38]. Such inconsistency of results between in vivo and in vivo results could also occur with OBD.

In recent years, machine learning (ML) techniques have been actively implemented to improve accuracy in the areas of medical diagnosis and screening. ML is a method of analyzing data in which a computer automatically discovers rules and patterns behind the data. And in the field of medical diagnosis, it is often used to detect diseases with high accuracy. In many applications in the field of radiological imaging, the performance of ML-based diagnostic systems has been shown to be comparable to the performance of a well-trained and experienced radiologist [47]. In addition, ML can easily merge various data as features at the same time, regardless of whether they are directly related to the disease or not, and is expected to increase the reliability of diagnosis. For example, Monte-Moreno used ML in the noninvasive estimation of blood glucose and pressure using photoplethysmograph and showed that variables that alone do not contribute to prediction, such as age and weight, can be merged with photoplethysmograph data to improve prediction accuracy [27].

The combination of OBD and ML techniques to predict osteoporosis may be sufficiently reliable for use in screening. The purpose of this study was to verify the feasibility of using the combination of OBD and ML techniques as a screening tool for osteoporosis.

## Materials and Methods

### Participants

The protocol for this study was approved by the Human research ethics committee of the Chulabhorn research institute. There were 203 participants (171 women and 32 men). Of the participants, 182 were patients who visited Chulabhorn Hospital to have DXA scans, and the remaining 21 were hospital staff. Age, sex, weight, height, and body mass index (BMI; kg/m^2^) were obtained from these participants, in addition to DXA and optical device measurements. Eleven healthy volunteers (10 males and 1 female, age range 22–30 years) were measured for 20 days to derive the coefficient of variation (CV) of the optical system. Informed consent was obtained from all participants who participated in the study.

### Measurement

203 participants underwent BMD measurements of the lumbar spine, femur, and forearm using DXA (iDXA, GE Healthcare, the USA). Results were expressed in terms of bone mineral content per square centimeter (g/cm^2^) and T-Score. According to the World Health Organization (WHO) definition of osteoporosis, participants were classified into osteoporosis (T-score ≤  − 2.5) and others based on the T-score of the lumbar spine and femur [6, 20]. All T-scores were automatically generated by iDXA. If the T-score values for the lumbar spine, femoral neck, and total hip of a single participant resulted in different classifications, the lowest classification was always applied.

**Fig. 1:**
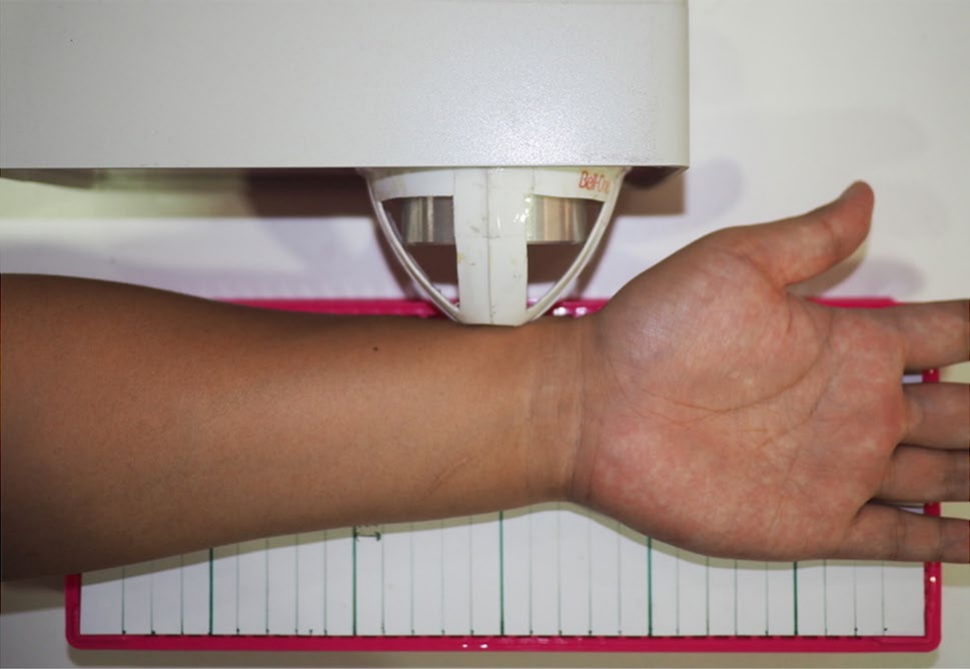
Measurement site of forearm. On the top in the photo is the optical bone densitometry device, measuring the ultra-distal radius

Optical measurements were carried out by trained staff immediately after the DXA. The ultra-distal (UD) radius of the non-dominant arm (10 mm proximal to the ulnar head) was measured in cross-sectional orientation with the palm side up, as shown in Fig. 1.

**Fig. 2:**
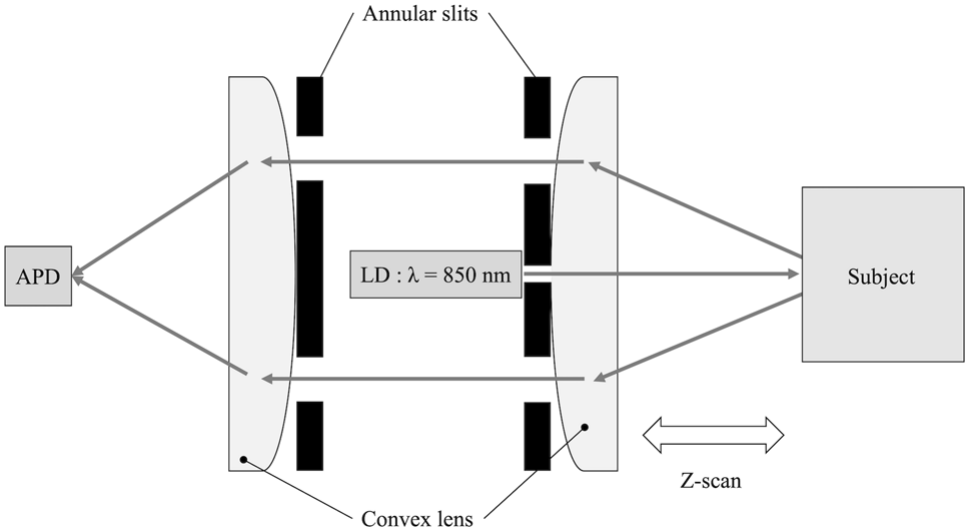
An optical bone densitometry system. APD: avalanche photodiode, LD: 850 nm wavelength 1mW laser diode [24]

The optical system consists of two convex lenses, two annular slits, a photodetector, and a laser diode, as shown in Fig. 2 [24, 25]. An avalanche photodiode (APD) module (C12703-01, Hamamatsu Photonics K.K., Japan) was used as the photodetector, and the analog light intensity data was converted to 10-bit digital data. The laser diode module used was 850 nm wavelength, 1 mW (H838501D, Egismos Technology Corporation, Canada). In this optical system, by moving the system in the direction of the measurement target, the light intensity distribution, spatially resolved in the radial direction from the light irradiation position, is acquired. The light intensity distribution was expressed in terms of the light intensity normalized by log_10_(*I/I*_0_) and the distance Z that the optical system traveled in the target direction from the lens focal length. Here, *I* is the detected light intensity and *I*_0_ is the incident light intensity. In this study, the slope of the light intensity distribution (SLID) was measured in the Z range of 35–42 mm. This range of SLID has been shown in previous research to be correlated with BMD [24, 25]. To detect unintended excessive target movement during measurements and eliminate improper measurement results, the light intensity distribution was measured twice in one measurement. The squared error ΔSLID of the slope of the light intensity distribution due to the motion of the measurement target is as follows:

1 $$\Delta {\text{SLID}} = \left( {{\text{SLID}}_{{\text{a}}} - {\text{SLID}}_{{\text{b}}} } \right)^{2}$$

where SLIDa and SLIDb are the SLIDs measured in the forward and backward optical system, respectively. Outliers were obtained using Tukey's boxplots for the distribution of all ΔSLIDs [41]. That is, the outliers and their thresholds are as follows:

2 $$\Delta {\text{SLID}} > {\text{SLID}}_{{{\text{th}}}}$$

3 $${\text{SLID}}_{{{\text{th}}}} = 75{\text{th percentile}} + 3\, {\text{IQR}}$$

where SLIDth is a threshold value of outliers, and IQR is interquartile range of boxplots.

### Machine Learning

The ML was used to predict an individual's minimum T-score as determined by DXA. The features for predicting T-score were selected from six variables: SLID, height, weight, BMI, age, and sex. For six variables, T-scores were predicted for two or more combinations (57 combinations) using each ML algorithm to derive the combination of features that yielded the highest area under the curve (AUC) of the receiver operating characteristic (ROC) for osteoporosis discrimination. The AUC takes values from 0.5 to 1, and an AUC of 1 indicates that the test is completely accurate for distinguishing between individuals with and without the condition of interest, while an AUC of 0.5 indicates that the test is useless.

Four different ML techniques were tested: Ridge Regression (RR) [15, 16], Support Vector Machines (SVM) [46], Random Forest (RF) [4], and Gradient Tree Boosting (GTB) [39]. RR solves a regression model where the loss function is a linear least squares function, and the regularization is given by the l2-norm [15, 16]. The RR was tested by varying the coefficients of the regularization term, which is included in scikit-learn 0.24.2 in python. SVM is insensitive to noise by using an ε-insensitive loss function, and nonlinear functions can be constructed by using kernel tricks [46]. The kernel chosen was a Gaussian kernel, and the kernel coefficients γ, soft margin C, and tube ε were varied for the tests. RF is one of the decision tree-based ensemble methods, where the output is the aggregated output of a set of classification or regression trees [4]. For RF, three parameters were varied and adjusted [34]; the number of decision trees in the ensemble, the minimum number of samples for a node to be considered a leaf, and the number of features to consider when computing the optimal node split. The RF used the one included in scikit-learn 0.24.2 in python. GTB is one of the ensemble learnings with decision trees and is a generalization of Boosting for arbitrary differentiable loss functions [10, 11]. Three parameters were varied in GTB; regularization factor, maximum depth of the tree, and the step size reduction used during the update to prevent overfitting. GTB used CatBoost 0.24.3.

**Fig. 3:**
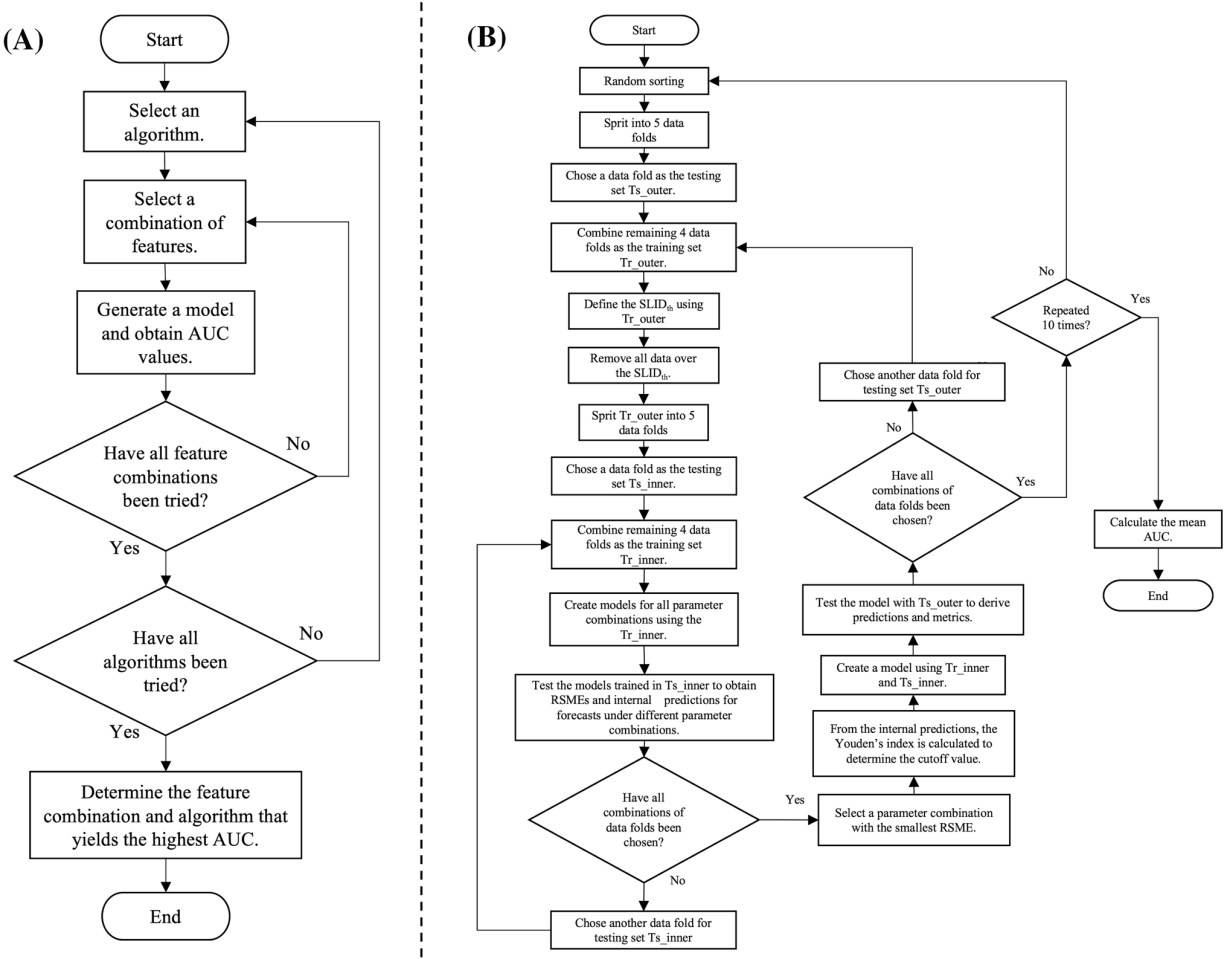
**A** Flowchart of the ML model determination procedure. **B** Flowchart of a ML model generation and validation

A flowchart of machine learning model generation is shown in Fig. 3. For each feature combination, the performance of the T-score predicted by each ML module to discriminate osteoporosis was validated by fivefold cross-validation. In the fivefold cross-validation, all data were first randomly divided into 5 equal-sized subsets, with one subsample as test dataset and the remaining 4 subsample as training dataset. To reduce selection bias in random sampling, fivefold cross-validation was repeated 10 times and the results were derived as an average. Each of the 50 training datasets in the 10 iterations of the fivefold cross-validation was trained with the following four steps. First, the outlier threshold SLID_th_ was determined using Equations 1–3 to detect and eliminate unintended excessive target movement during the measurement. Second, the best combination of parameters for a ML module were selected by a grid-search with fivefold cross-validations. Grid-search is a method that comprehensively searches for specified parameter candidates and selects the optimal parameter set. The optimal combination of parameters was assumed to yield the minimum value of the root mean squared error (RSME). Third, from the predictions obtained by grid search with fivefold cross-validation, an ROC curve was derived and a cutoff value for the T-score to classify osteoporosis was determined using Youden's index. Youden's index selects the cutoff value that maximizes the sensitivity + specificity − 1 [49]. Finally, once the best set of parameters was found, the ML module was retrained with all the data in the fold. This method of adjusting the model using cross-validation within each fold of cross-validation is commonly referred to as nested cross-validation [21], and allows for the verification of model performance, including the derivation process.

### Statistical Analysis

Student's t-test and Pearson's chi-square test were used to compare the characteristics of the osteoporosis and non-osteoporosis groups. Pearson's correlation coefficient was used to investigate the association between SLID and age, BMD, and T-score. To evaluate the osteoporosis discrimination performance of the ML model, ROC analysis was performed, and AUC was calculated. The mean, standard deviation, median, first and third quartiles of cutoff value, accuracy, sensitivity, specificity, positive predictive value (PPV), and negative predictive value (NPV) for classifying osteoporosis were also determined. Wilcoxon signed-rank test was performed to compare the performance differences of the proposed ML model with and without SLID. All statistical analyses were performed with SciPy 1.8.0 in Python 3.8.

## Results

**Fig. 4:**
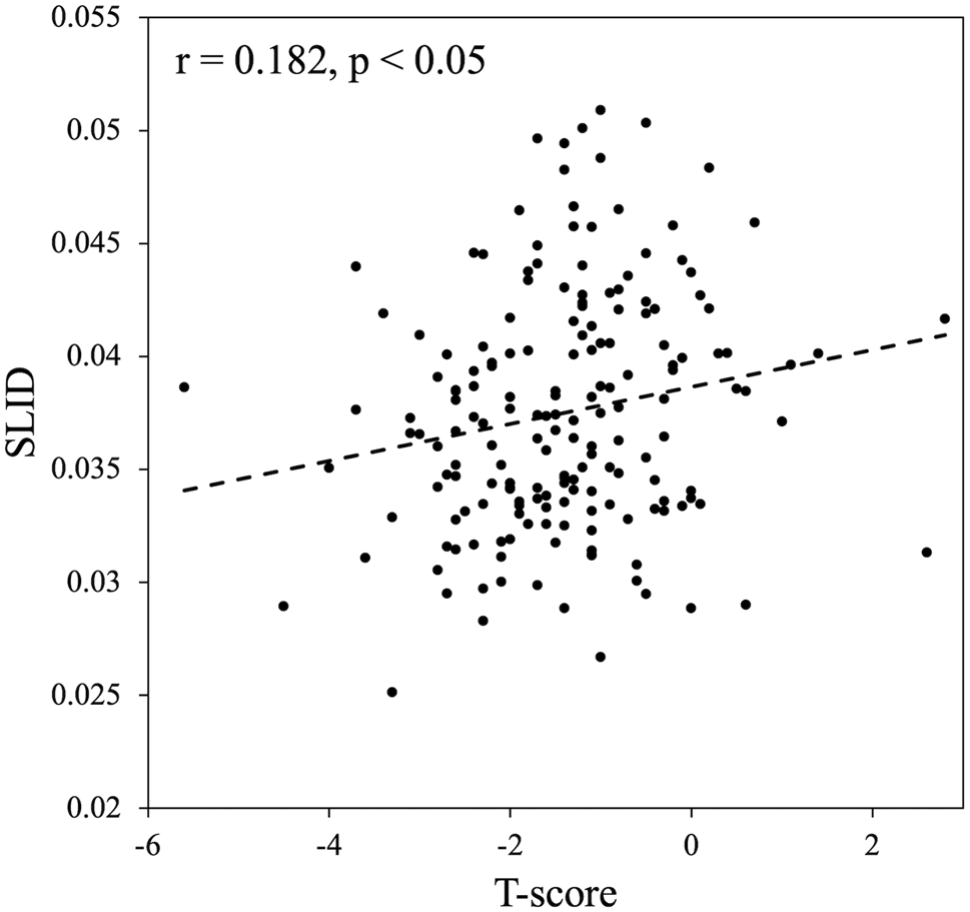
Relationship between SLID and T-score determined by DXA

A total of 203 Thai people aged between 22 and 96 years (mean age: 64.7 years) were included, and 84.2% were woman in this study. The prevalence of osteoporosis (T-score ≤ − 2.5) was 18.2%. As expected, the participants with osteoporosis had a higher average age, shorter height, and lower weight than the non-osteoporotic participants. SLID before outlier removal did not differ between the groups, but as shown in Table 1, SLID after outlier removal was significantly lower in the osteoporosis group than in the non-osteoporosis group; the CV of SLID was 3.77 ± 1.91 %, and the measurements were stable. SLID and T-score determined by DXA showed a weak correlation of *r* = 0.182 (Fig. 4). Here, T-score is the minimum value obtained for the lumbar spine, femoral neck, and total hip.

**Table 1:** Demographic

	Total (*n* = 203)		Non-osteoporosis (*n* = 166)		Osteoporosis (*n* = 37)		*p* value
	Mean	SD	Mean	SD	Mean	SD	
Age [years]	62.8	14.8	62.1	14.0	73.3	10.5	< 0.001
Weight [kg]	60.6	10.0	64.1	9.6	49.9	9.1	< 0.001
Height [cm]	158.0	7.3	160.1	7.6	153.6	7.7	0.001
BMI [kg/cm^2^]	24.3	3.8	25.0	3.5	21.2	3.6	< 0.001
Number of woman (n [%])	171 (84.2 %)		137 (82.5 %)		34 (91.9 %)		0.158
UD radius BMD [g/cm^2^]	0.358	0.088	0.417	0.075	0.285	0.052	< 0.001
T-score	− 1.43	1.16	− 0.21	0.75	− 3.04	0.63	< 0.001
SLID (n)	0.0375 (180)	0.0053	0.0386 (61)	0.0055	0.0354 (30)	0.0042	< 0.05
Original SLID	0.0373	0.0057	0.0382	0.0065	0.0358	0.0042	0.086

The values are given as the mean and standard deviation. SLID was removed outlier by Eq. 1, and the original SLID shows the data before the outliers were removed. The number of participants was listed at the top of the table unless otherwise indicated. *p* values were derived from the unpaired *t* test or *χ*^2^ test for the difference between osteoporosis and non-osteoporosis group

*SD* standard deviation

The RR model with SLID, age, and weight as features was selected as the osteoporosis prediction model. The criteria for selecting the machine learning algorithm and features were the AUC value of the predicted T-score for osteoporosis discrimination. The performance of the model was tested by repeating the nested fivefold cross-test 10 times against a database of 203 individuals, excluding SLID outliers. That is, 80% (162) of the randomly selected databases were used to train the model, leaving 20% (41) as the test database, which was rotated 5 times for 10 iterations. In 80% of the databases used for training, thresholds were set to define SLID outliers, and the best structure, parameters, and cutoff values were determined using five-part cross-validation. These nested cross-tests, including the derivation process, estimate the generalization performance of the predictive model, and their repetition reduces the selection bias of random sampling. For all algorithms, SLID, age, and weight were selected as the features with the highest AUC. RR showed the highest AUC for osteoporosis discrimination.

**Fig. 5:**
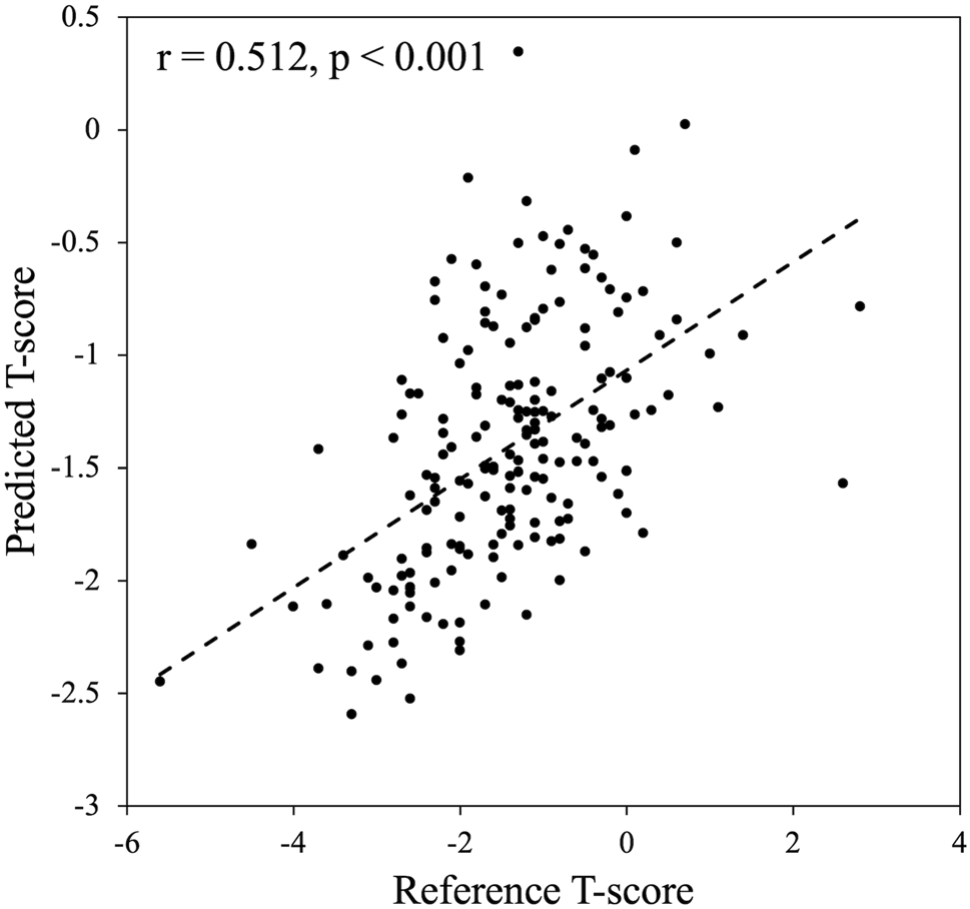
Relationship between reference and predicted T-score by RR model

**Fig. 6:**
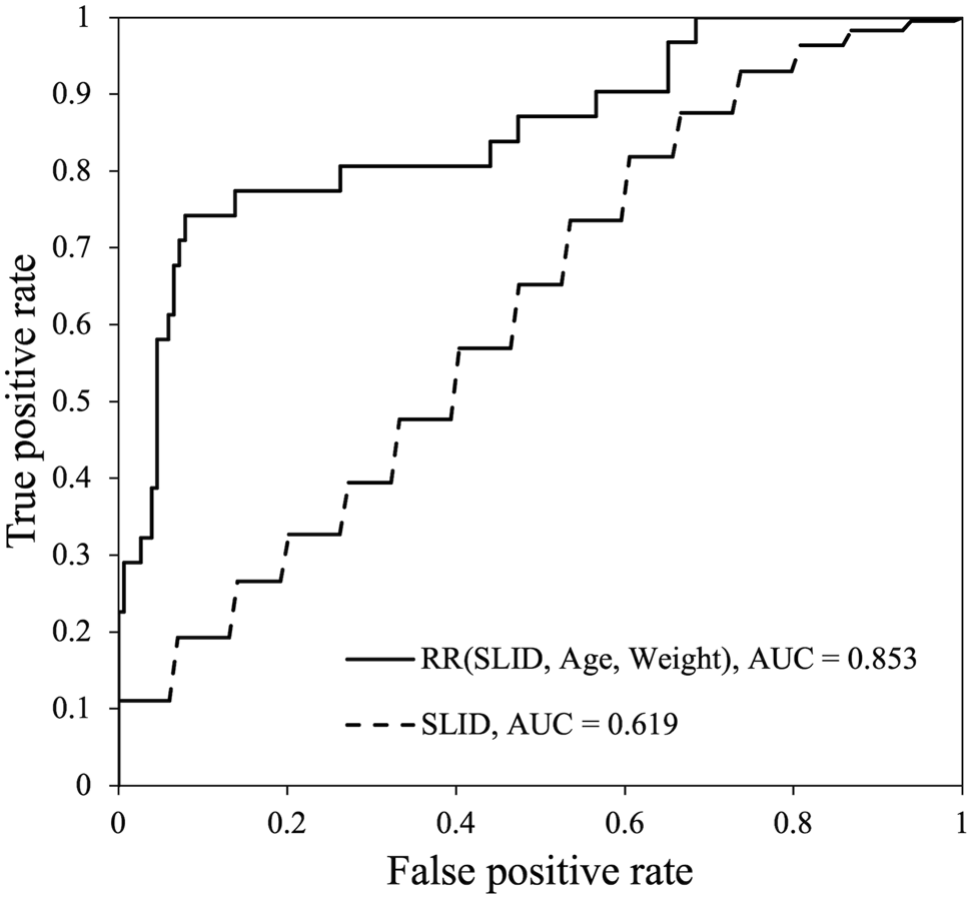
ROC curves comparison between RR model and SLID

A comparison of the T-score predicted using the RR module and SLID, age, and weight with its reference value is shown in Fig. 5. The predicted T-score had a correlation of *r* = 0.512 with respect to the reference value, showing a higher correlation coefficient compared to the correlation between SLID and T-score (*r* = 0.182). Figure 6 shows the ROC curves for osteoporosis discrimination obtained from the T-score predicted by RR (Fig. 5) and the SLID value (Fig. 4). On the other hand, the AUC of T-score predicted by RR was 0.853. The performance of osteoporosis discrimination was greatly improved by using the machine learning technique. Generally, an AUC of 0.80 to 0.90 is considered to have high discrimination performance, 0.70 to 0.80 is considered moderate, and 0.60 to 0.70 is considered low [29]. Table 2 shows comparison of the classification performance by RR with and without SLID. The cutoff values determined from the Youden index in the internal cross-validation did not differ significantly between the two models. The accuracy, sensitivity, specificity, PPV, and NPV determined from the cutoff values were significantly higher in the model with SLID compared to the model without SLID: 4.9% for accuracy, 9.0% for sensitivity, 4.4% for specificity, 13.4% for PPV, and 13.4% for NPV for the model with SLID compared to the model without SLID. PPV 13.4 %, and NPV 1.3 % higher. These results indicate that SLID contributes to the improved osteoporosis discrimination performance of machine learning models.

**Table 2:** Comparison of prediction performance by RR with and without SLID

	RR (SLID, age, weight)					RR (age, weight)					*p* value
	Mean	SD	Median	Q1	Q3	Mean	SD	Median	Q1	Q3	
AUC	0.853	0.099	0.875	0.790	0.930	0.843	0.100	0.864	0.776	0.926	< 0.01
Cutoff	− 1.886	0.056	− 1.876	− 1.857	− 1.925	− 1.848	0.104	− 1.866	− 1.761	− 1.925	0.08
Accuracy	0.875	0.056	0.867	0.842	0.918	0.834	0.104	0.836	0.796	0.889	< 0.001
Sensitivity	0.703	0.205	0.714	0.579	0.857	0.645	0.200	0.667	0.500	0.800	< 0.01
Specificity	0.909	0.058	0.927	0.867	0.965	0.871	0.097	0.895	0.802	0.937	< 0.001
PPV	0.628	0.187	0.625	0.500	0.750	0.554	0.218	0.500	0.400	0.714	< 0.001
NPV	0.940	0.038	0.936	0.918	0.964	0.928	0.035	0.930	0.897	0.958	< 0.001

Q1 and Q3 are the first and third quartiles in the quartile range

*SD* standard deviation

## Discussion

The purpose of this study was to verify the feasibility of using the combination of OBD and ML techniques as a screening tool for osteoporosis. To predict T-score, an RR model was generated using SLID which is measured by OBD, age, and weight as features. The predicted T-score showed an AUC of 0.853 against identification of osteoporosis.

The ML model generated in this study has the potential to work in terms of operational and reliability of osteoporosis screening. OBD was developed as a simple method of measuring BMD, and age and weight demographic data are readily available. The OBD can take measurements in seconds by simply holding up an arm, and age and weight demographic data are readily available. If it can maintain good performance after being validated in a larger population, the ML model proposed in this study may be used as one of the pre-screening methods for osteoporosis. According to a meta-analysis by Nayak et al, the AUC of QUS for osteoporosis discrimination is approximately 0.74 for speed of sound and 0.77 for broadband ultrasound attenuation [30]. In a study by Boonen et al. the AUCs discriminating osteoporosis in DXR and RA were 0.84 and 0.80, respectively [3]. Considering that these methods are commonly used for screening purposes, it is suggested that our model (AUC = 0.853) may have adequate performance.

Linear regression models such as OST was developed to screen for osteoporosis in a simplified manner based on age and weight. The RR algorithm generates a linear regression model. In this study, the AUC to discriminate osteoporosis from T-score predicted from age and weight only using the RR algorithm was 0.843. These results are within the range of 95% confidence intervals for AUC in OST [17, 48] which support the results obtained in this study.

The criteria for feature and algorithm selection were the AUC score of the predicted T-score for osteoporosis discrimination. Age and weight, as used in OST, are known to be useful in predicting osteoporosis, and these indicators were also important in this study. Sex is one of the well-known risk factors for osteoporosis, but it was not selected in our model. This result is consistent with previous reports, as most of the dataset is likely to be female [18]. Although RR, a linear regression model, performed better than SVM, RF, and GTB, which can also be used for nonlinear problems, linear predictors were not chosen as the final model in several previous studies [34, 48]. For example, in the study by Yoo et al. SVM was selected as the final model instead of logistic regression, a linear classifier, to predict osteoporosis [48]. The reason why RR showed the best performance in this study could be due to the selection of features from a small number of variables. In this model, features were selected from a limited set of six variables, whereas in the Yoo’s model, features were selected from 15 variables. While more sophisticated algorithms may be selected in the future as more functionalities becomes available and the nonlinearity between dependent and independent variables increases, fewer variables have the advantage of simplifying the model and requiring less input effort.

Osteoporosis discrimination performance of SLID alone was not sufficient. This could have several causes. The first is because the measurement target of OBD is the distal radius. The distal radius does not show a strong correlation to BMD of the femur and lumbar spine [22, 28], although it is one of the most frequent fracture sites in osteoporosis [26]. The WHO definition of osteoporosis we used is based on BMD of the femur and lumbar spine. The second is the effect of the soft tissue layer. Most bone matrix in body is at least covered by soft tissues such as epidermis, dermis, and subcutaneous tissue. However, our previous phantom experiments assumed a monochromatic, homogeneous light scatterer composed of one layer as soft tissue, although SLIDs showed a strong correlation to BMD [24, 25]. There is concern that the measurements may also be affected by the complexity of the morphology of the soft tissue layer. Pifferi et al. measured human calcaneus using time-resolved transmission spectroscopy, suggesting that the complexity of the soft tissue covering the bone and differences between subjects can lead to large measurement bias [35]. However, in this study, SLID combined with ML techniques significantly improved osteoporosis discrimination performance. This result was attributed to the addition of weight and age, information not available in the SLID measurement, which contributed to reducing the SLID measurement error. The results suggest that using ML techniques and adding some features can reduce the performance limitations of OBD for osteoporosis discrimination.

SLID was one of the important indicators in the ML model, although it is not accurate enough on its own to discriminate osteoporosis. The highest performing models in all ML algorithms tested included the SLID in their features. The RR model using SLID, age, and weight showed a significant improvement of 1.2% in AUC and 4.9% in accuracy compared to the model using only age and weight. Although the 4.9% improvement in accuracy may be numerically slight, if Thai women over the age of 50 are considered a population at risk for rapid bone loss, there are approximately 8.97 million women in the population that fall into this category, and 4.9% equates to approximately 440,000 women. In addition, all measures of sensitivity, specificity, PPV, and NPV improved by 1.3–13.4% with and without SLID.

We recognize several limitations regarding this study. First, the applicability of the model developed in this study is limited by the subject population. All participants in this study are Thai, and their physical characteristics, lifestyles, cultural backgrounds, and environmental living conditions differ from those of other populations. In addition, most participants were patients who came to the hospital for a DXA, so it is likely that many participants at potential risk for osteoporosis were included in the analysis. Although the derivation method of this model is promising, its implementation in clinical practice requires validation in an independent population and the setting of appropriate cutoff values for the intended purpose. Second, in this study, SLID was used for the actual measurements of the ML model, but screening instruments such as QUS could be used as well. However, OBD does not require the application of gel and allows for quick and simple measurements. ML models using OBD may offer a new option as a screening method for osteoporosis. Third, the distal radius is not a priority in the current diagnosis [6]. However, the forearm is one of the ideal sites for simple light-based measurements because it is easily exposed and has little soft tissue, especially in the UD area. In addition, a history of wrist fracture is associated with the risk of future fractures [2], and it has been noted that BMD measurement in the forearm may facilitate early detection of patients in need of intervention for osteoporosis [26]. Therefore, it may be a promising measurement location for early detection of osteoporosis.

The purpose of this study was to verify the feasibility of using the combination of OBD and ML techniques as a screening tool for osteoporosis. A RR model was developed to predict T-score using OBD measurements as well as age and weight. The results obtained suggest that the developed model is reliable enough to be used for screening for osteoporosis.

## Data Availability

The codes, data, and materials that support the findings of this study are available from the corresponding author upon reasonable request.

## Declarations

### Registration

Research project had been approved for registration at TCTR. TCTR Identification Number is TCTR20190911001, and registration date is 10th September 2019.
